# Increase in self-reported empathy during medical school training: A longitudinal study

**DOI:** 10.1371/journal.pone.0332343

**Published:** 2025-09-15

**Authors:** Nieves Díez, María Cristina Rodríguez-Díez, Leire Arbea, José Pereira, Fermín Rodríguez, Marcos Trelles, Alfredo Gea, Loreto García del Barrio

**Affiliations:** 1 Medical Education Unit, School of Medicine, University of Navarra, Pamplona, Spain; 2 New affiliation, Department of Health Science, Medicine, Public University of Navarre, Pamplona, Spain; University of Tabuk, SAUDI ARABIA

## Abstract

**Background:**

Empathy is a fundamental tool in clinical practice, but despite its importance and benefits, it is often underrepresented in medical curricula. This study explored the evolution of medical students’ empathy longitudinally across six years of undergraduate training following the introduction of a curriculum with 4-hour workshops during each of the six clinical rotations from Years 4 to 6, complemented by self-reflections after each rotation.

**Methods:**

Students of the 2016 class (graduated 2022) completed the Jefferson Scale of Empathy-Students (JSE-S) and the Interpersonal Reactivity Index (IRI) at the beginning of Year 1 (T0) of studies, beginning Year 3 (T1), end Year 3 (T2) and end Year 6 (T3). The NEO Five-Factor Personality Inventory was completed at T0. Empathy scores across timepoints were analyzed using repeated measured analyses of variance (ANOVA).

**Results:**

36.1% and 32.5% of 169 eligible students completed the JSE-S and IRI scores respectively at all four timepoints. The JSE-S total mean score increased significantly across the six years of medical course (p < 0.001), as did the IRI total mean score (p < 0.001). In addition, the mean JSE-S total score increased significantly between T3 vs T0 (p < 0.001), T3 vs T2 (p < 0.001 and T2 vs T1 (p = 0.028); and the mean IRI total increased significantly between T3 vs T0 (p < 0.001) and T3 vs T2 (p < 0.001).

**Conclusion:**

Medical students who experienced a new professionalism program increased their empathy as measured by the JSE-S and IRI instruments across the six years of curriculum.

## Introduction

Empathy is a fundamental tool in the development of therapeutic relationships between health care providers and the patients they care for [1,2]. It is a multifaceted concept, making a single definition elusive. Riess defines empathy as a ‘complex capability enabling individuals to understand and feel the emotional states of others, resulting in compassionate behaviour’ [3]. This definition encompasses two components of empathy, an affective and a cognitive component, and links it to compassion. While empathy is about awareness of other another’s emotions and trying to understand their feelings, compassion is about taking action in response to empathy [4]. Affective empathy may be viewed as a personal trait that creates emotional responses through the ability to relate, understand and join another person’s experiences, while cognitive empathy may be understood as a skill that can be acquired and nurtured through education, and understanding another’s experience need not evoke detrimental emotional responses [5].

Empathy on the part of care providers imparts numerous benefits, on patients and the care providers alike. Among others, it strengthens the doctor-patient relationship, increases patient satisfaction, improves treatment compliance and patient outcomes, increases professional satisfaction and mitigates burnout [1,2,6–9]. Patients identify it as a key requirement in health care [10]. Despite its importance and benefits, it is often underrepresented in medical curricula [11,12]. Moreover, there is evidence that unless explicitly addressed in curricula, medical training may unintentionally erode empathy [13–16].

Fortunately, there is growing evidence of retained or improved empathy and compassion on at least one outcome measure arising from education interventions in medical curricula that intentionally address these domains [12,17]. A variety of learning approaches have been employed to nurture empathy. These include group discussions and personal reflections on clinical and other experiences, simulated scenarios and role-playing, the use of the arts and humanities, and reflective writing [11].

Our group previously reported a cross-sectional study in which a cohort of final year medical students (graduating class of 2019) who had received a new multi-year (years 4–6), reflection-based curriculum designed to nurture empathy, compassion and professionalism [18], was compared to a control cohort of final year students (graduating class of 2018) who had not received that curriculum. Empathy scores, measured by standardized patients using the Consultation and Relational Empathy (CARE) scale and the Jefferson Scale of Patient Perceptions of Physician Empathy (JSPPPE), were significantly higher in the intervention group than the control group. However, no significant difference was found between the two groups when empathy was self-assessed by students using the Jefferson Scale of Empathy-Students and the Interpersonal Reactivity Index [19].

The aim of this longitudinal study was to explore the evolution of empathy across six years of medical training at the University of Navarra in Spain, in a cohort of medical students who received a new longitudinal multi-year education program (called “Itinerario de Identidad Médica” or “Medical Identity Journey”) designed to intentionally nurture empathy, compassion, professionalism, integrity, communication, ethical decision-making, and teamwork in the school’s undergraduate medical curriculum. These themes are considered by our school as key aspects of medical identity.

## Methods

### Intervention

The “Medical Identity Journey” program, introduced in 2013, is an obligatory transversal program that is offered in the fourth, fifth and sixth (final) years of study [18]. It focuses on themes related to medical identity, including empathy and compassion, professionalism, integrity, communication, leadership and teamwork, and commitment to the patient. Each of the educational interventions (two per year) consists of three components: 4-hour-long facilitated workshops, formal written reflections on the themes by students on experiences during their clinical rotations, and formative feedback from clinical tutors on their reflections.

The first workshop in the first semester of the fourth year focuses on empathy and compassion. It is designed to be highly interactive with student participation through small and large group discussions. It uses the humanities (works of art, clips from movies such as “Wit” and images), patient testimonials and cases to elicit reflections, discussions and learning, as well as short theoretical overviews of the topic. There are about 210 medical students a year at the school, and to enhance learner interactivity and engagement in these workshops, students are divided into three equal groups of seventy. Each group takes turns participating in the workshop (the workshop is therefore repeated three times). After the workshop, during their clinical rotation, they are asked to intentionally look out for moments during patient care in which empathy and compassion are shown or are absent and to reflect on these. Towards the end of that rotation, they have to select one of those experiences and write a reflective essay of up to 500 words about that experience. Following this, students meet with their clinical tutor to discuss their reflections. During this meeting, the tutor offers formative feedback, helping the student deepen their understanding of the experience and its implications for their professional development. Once this discussion has taken place, students upload their written reflections to an online learning management system. The workshop facilitator then reviews and assesses the reflections using a standardized rubric. This rubric includes aspects such as the depth of reflection, the personal impact of the experience on the student, and how the experience and lessons learned will influence their future patient care. Additionally, the facilitator provides each student with individualized feedback on their reflection.

This process is repeated during each of the five subsequent clinical rotations from years 4 to 6, resulting in six workshops. Consequently, each student completes six written reflections, receives six individualized formative feedback sessions from clinical tutors, and undergoes six summative assessments by the workshop facilitator based on the aforementioned criteria.

Given the influence of role modeling on learners with respect to empathy and other aspects of medical identity [15], clinical supervisors are also encouraged to role model these qualities when the students are undertaking their clinical rotations.

### Overall study design, participants and recruitment

This was a prospective longitudinal study of undergraduate medical students who started their training in the 2016–2017 academic year and graduated in 2022. Participation was voluntary and confidential.

### Outcome measures and data collection

After providing written informed consent, participants completed a demographics questionnaire at baseline (start of the first year of studies), followed by three instruments: the Jefferson Scale of Empathy-Students (JSE-S), the Interpersonal Reactivity Index (IRI) and the Neuroticism, Extraversion, Openness Five-Factor Inventory (NEO-FFI). We opted to use two instruments (JSE-S and IRI) to assess empathy given the complexity of the construct and the limitations of any one single instrument to assess the construct. Zhou et al. have recommended the use of two or more instruments when study empathy [11]. These two instruments and the NEO-FFI are designed for self-rating by study participants and validated Spanish (Spain) versions were used in this study [20–22].

The JSE-S scale, developed specifically for medical education, contains 20 Likert-type items with seven-point scales (1 = strongly disagree to 7 = strongly agree) [23,24]. It includes three subscales: “Perspective Taking” (JSE-S PT; 10 items), “Compassionate Care” (JSE-S CC; 8 items) and “Standing in the Patient’s Shoes” (JSE-S STS; 2 items). The instrument prioritizes the cognitive component of empathy. The IRI was originally designed for a general population and contains 28 items, each rated using a five-point Likert scale (1 = does not describe me well to 5 = describes me well) [25]. The scale consists of four 7-item subscales: “Perspective Taking” (IRI PT), “Fantasy Scale” (IRI FS), “Empathic Concern” (IRI EC), and “Personal Distress” (IRI PD). The focus is on the cognitive and emotional components of empathy.

The NEO-FFI instrument explores personality traits across five dimensions, namely Neuroticism, Extraversion, Openness, Agreeableness, and Conscientiousness. It consists of 60 items, each assessed using a five-point Likert-type scale [26].

**Fig 1:**
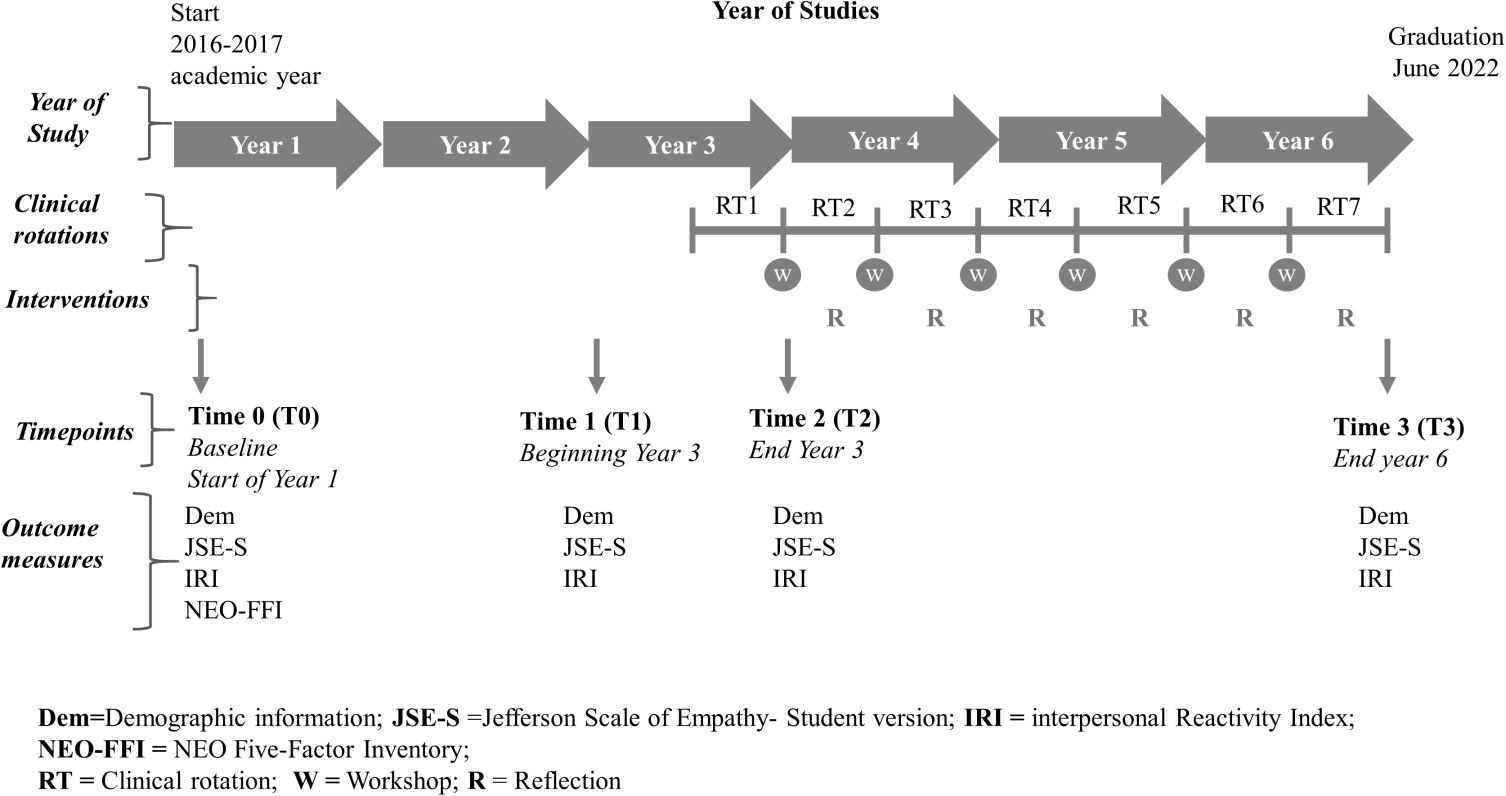
Study timelines: data collected at timepoints and context of intervention.

Data collection occurred at four points in time (see Fig 1): baseline at the beginning of the first year of studies (T0, September 22, 2016); the beginning of Year 3 (T1, September 10, 2018), the end of Year 3 (T2, March 29, 2019) and the end of the sixth and final year of study (T3, February 28, 2022). At baseline, demographic information was collected, and participants completed JSE-S, IRI and NEO-FFI questionnaires. The JSE-S and IRI instruments were then collected at T1, T2 and T3. See Fig 1 for a summary of the timelines and their timepoints of the study, the data collected at each timepoint and the timing of the intervention across the six years of study.

Demographic data included age, gender, personal experiences of serious illness (themselves, family members or friends), volunteer experience, preferred elective rotations (there are several rotations that students may choose from) and their preferred specialties following undergraduate training. Specialty preferences and medical rotations were grouped into medical (including the medical specialties, family medicine and psychiatry) and non-medical (including surgical, imaging and anesthesia). Each of the assessment timepoints were opportunities to update demographic information that may have changed following baseline assessment.

### Analyses

Descriptive statistics were used to analyze participants’ demographics and characteristics. Repeated measures analysis of variance (ANOVA) with Bonferroni Correction (Bonferroni Post Hoc Test) was used to compare the mean results of the outcome measures (JSE-S and IRI) across the four timepoints. Effect sizes, to determine the magnitude and practical significance of the differences, were calculated using the partial eta squared (η²p) measure.This is a preferred measure in the context of ANOVA analyses. Values around 0.01 indicate a small effect size, around 0.06 a medium effect, and around 0.14 or more a large effect [27].

Comparisons of empathy scores were also undertaken between pairings of timepoints; the paired t-test was used.

A multivariate analysis using multiple regression was performed to explore mean changes in JSE-S and IRI scores (and their respective subscales) across different timepoints (specifically T3 versus T0, T2 versus T1 and T3 versus T2) and different factors related to empathy. The dependent variables were the JSE-S or IRI empathy changes, and the independent variables included age, gender, personal experiences of illness, volunteer experience, preferred specialization, number of clinical rotations and personality trait.

Only those study participants who had completed the instruments at all four timepoints were included in this analysis. A comparative analysis was undertaken of the baseline characteristics and scores between students who participated at all four timepoints versus those who did not. Continuous variables were compared using the Student’s t-test and categorical variables with the chi-square test.

The statistical significance threshold was set at 0.05 for all statistical tests performed (except Bonferroni post hoc test), and all were two-tailed. Internal reliability was calculated for the instruments using Cronbach’s alpha coefficient. All statistical analyses were performed with STATA software (16.1, StataCorp LLC, College Station, TX).

### Ethics

The study was approved by the Research Ethics Committee of the University of Navarra (project 2016.025).

## Results

### Demographics

A total of 169 of the 205 students in the class (82.4% of students) volunteered to participate in the study and completed all the outcome measures at baseline (T0). Of these 169 students, 61 (36.1%) and 55 (32.5%) completed the JSE-S and the IRI respectively for all four timepoints (T0, T1, T2, and T3). The response rate for each of the timepoints was 82.4% (169/205) for T0, 64.4% (132/205) for T1, 58.5% (120/205) for T2 and 54.9% (112/204) for T3.

Participant’s demographics are shown in Table 1. At T0, 59.2% of the participants identified themselves as women and the mean age was 18.2. Just over two thirds of students had done volunteer work. Preference for a medical specialty increased across the timepoints, from 52.5% at T0 to 77.1% at T3, as did the experience of a serious illness in someone close to them (37% at T0 to 52% at T3).

**Table 1:** Study participant demographics.

	T0 (*N* = 169)	T1 (*N* = 132)	T2 (*N* = 120)	T3 (*N* = 112)
Gender: Female	100 (59.2%)	82 (62.1%)	71 (59.2%)	66 (58.9%)
Gender: Male	69 (40.8%)	50 (37.9%)	49 (40.8%)	46 (41.1%)
Age: Mean	18.2	20.2	20.9	24.2
Own serious illness: Yes (%)	7 (4.2%)	6 (4.7%)	7 (5.9%)	4 (3.6%)
Serious illness of someone close: Yes (%)	60 (37.0%)	76 (59.8%)	65 (55.1%)	64 (58.2%)
Volunteerism: Yes (%)	112 (66.7%)	88 (68.8%)	82 (68.3%)	71 (64.5%)
Specialty preference: Medical	83 (52.5%)	79 (62.2%)	84 (71.2%)	84 (77.1%)
Specialty preference: Non-medical	75 (47.5%)	48 (37.8%)	34 (28.8%)	25 (22.9%)
Medical internship: Medical specialty			62 (51.7%)	
Medical internship: Non-medical specialty			58 (48.3%)	
Medical internships: ≥ 5 medical specialties				68 (62.4%)
Medical internships: < 5 medical specialties				41 (37.6%)

T0: Baseline (beginning of first year); T1: Beginning of third year; T2: End of the third year (after the first clinical rotation); T3: End of final year of studies.

### Internal reliability of instruments

Cronbach’s Alpha coefficients were greater than 0.7 across all the time periods (T0, T1, T2, T3) for both the JSE-S and the IRI, indicating good internal consistency. For the JSE-S the coefficients were 0.729, 0.735, 0.757 and 0.805 for T0, T1, T2 and T3 respectively, and for the IRI they were 0.786, 0.766, 0.776 and 0.709.

### Empathy across timepoints

**Fig 2:**
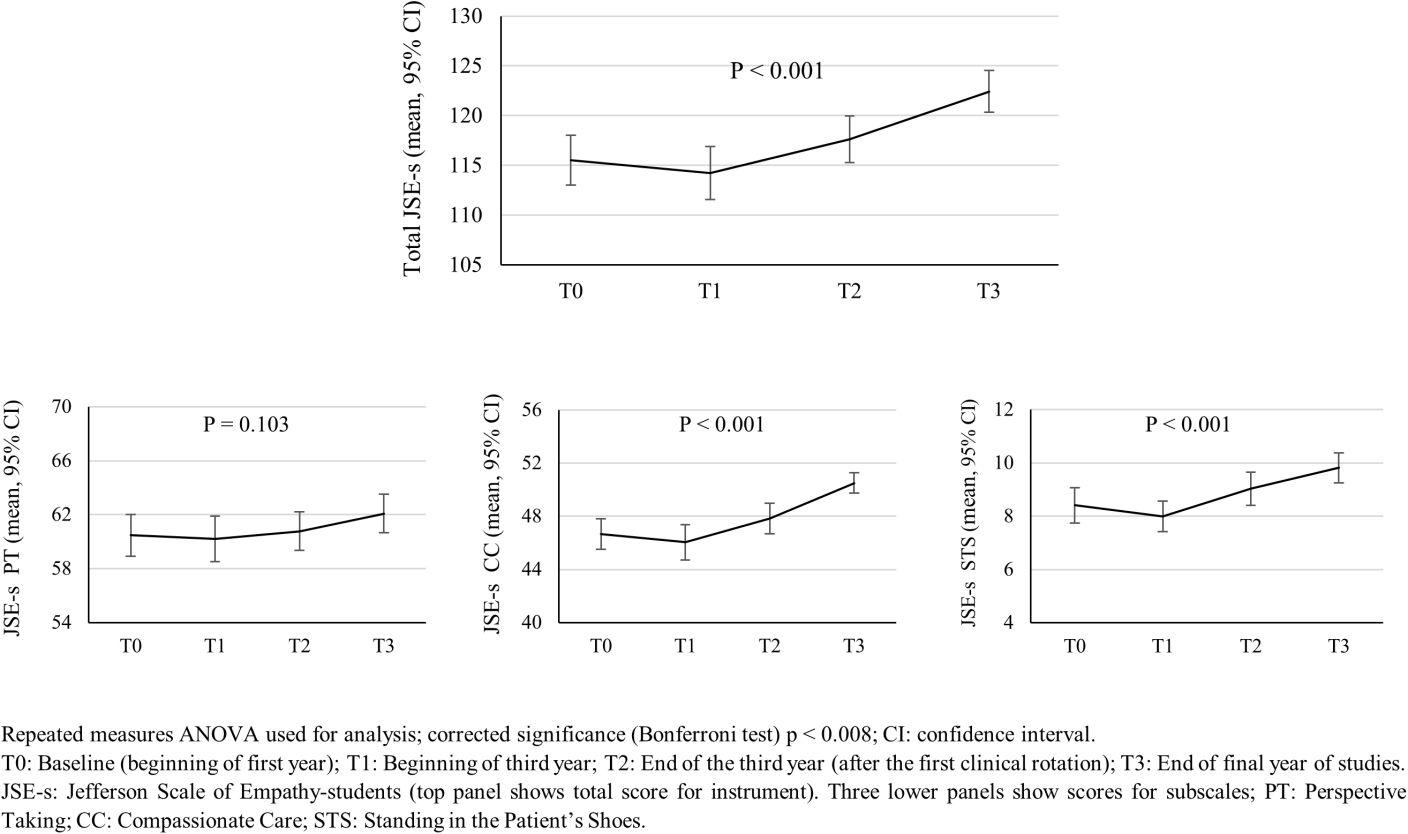
Mean scores of the Jefferson Scale of Empathy for Medical Students (JSE-S) and confidence intervals across the four timepoints (T0, T1, T2 and T3); total JSE-S score and scores by subscales (*N* = 61 students).

For students who submitted data at all four time points, the JSE-S total mean score and the mean scores of the JSE-S CC and JSE-S STS increased significantly across the timepoints (see Fig 2); for the total score: F (3, 240) = 15.94, p < 0.001 and for the subscales JSE-S CC: F (3, 240) = 17.54, p < 0.001 and JSE-S STS: F(3, 240) = 10.30, p < 0.001. Fig 2 shows the increase in mean scores across the timepoints, specifically increases from the T1 to T3 timepoints; there was no increase in the mean total and subscale scores between T1 and T0.

**Fig 3:**
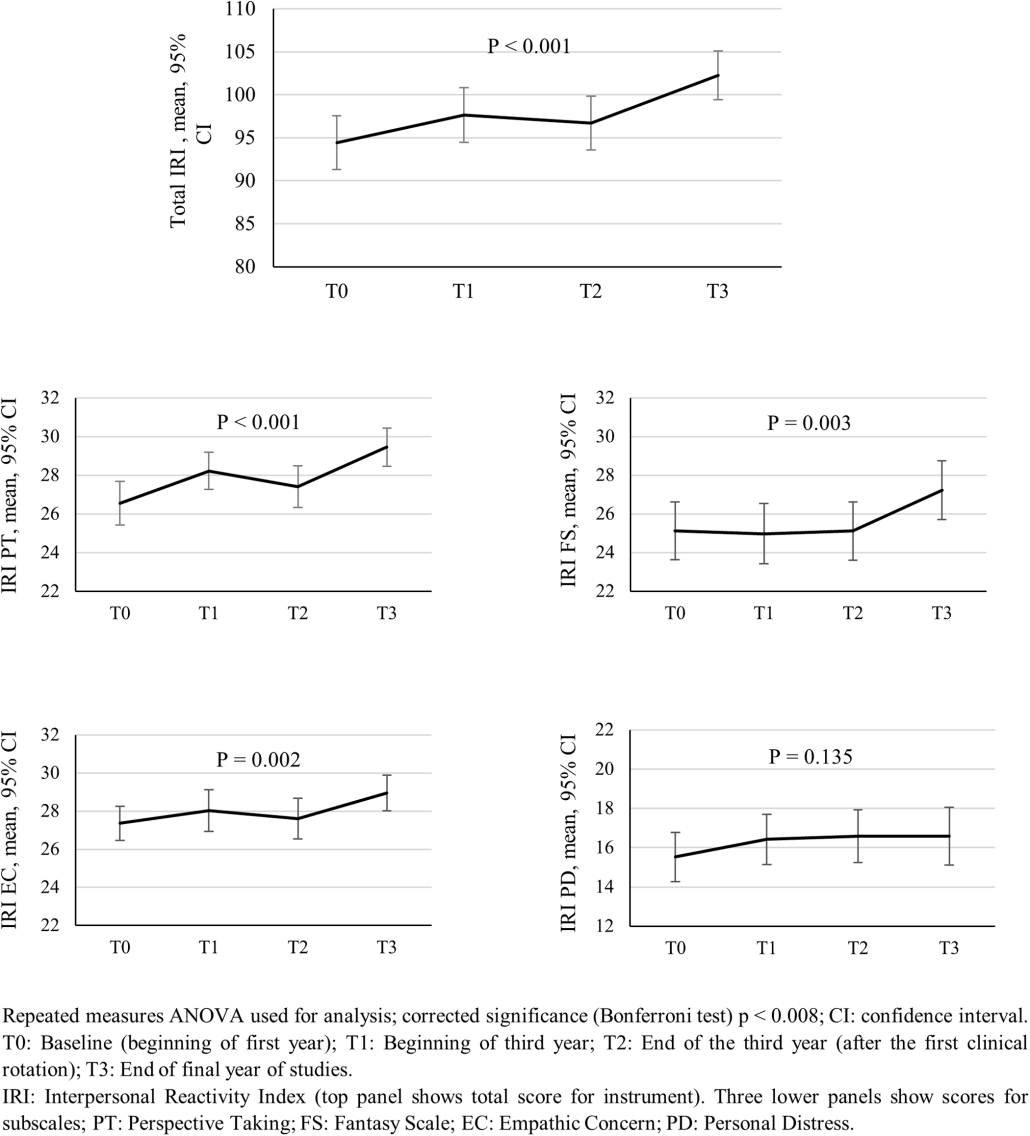
Mean scores of the Interpersonal Reactivity Index (IRI) and confidence intervals across the four timepoints (T0, T1, T2 and T3); total IRI score and scores by subscales (*N *= 55 students).

Significant increases in mean scores were also observed in the IRI total and its IRI PT, IRI FS and IRI EC subscales (see Fig 3); for the total score F (3, 216) = 13.72, p < 0.001 and for the subscales IRI PT: F (3, 216) = 11.66, p < 0.001; IRI FS: F (3, 216) = 5.34, p = 0.003; IRI EC: F (3, 216) = 5.33, p = 0.002. Fig 3 shows that the increase in mean scores across the timepoints is largely due to increases during the T2 to T3 timepoints. There were no significant increases in the IRI PD subscale scores.

Table 2 shows the results of the empathy scores between the timepoints. For the time periods T3 (end of sixth year) versus T0 (beginning of first year), significant increases were noted in the total and the subscale scores for both instruments, except for the IRI PD subscale. Between T2 (end of third year) and T1 (beginning of third year), during which the first clinical rotation was done but no workshop presented, significant increases in mean scores were found in the JSE-S total, JSE-S CC and the JSE-S STS subscales. No increases were found in the IRI total or subscales scores during this time period. In the T3 (end of sixth year) versus T2 (end of third year) timepoints analysis, when the majority of clinical rotations were held and the “Medical Identity Journey” curriculum presented, significant increases were noted in the total scores of both instruments and for most of the subscale scores, except for the JSE-S CC and IRI PD subscales (see Table 2).

**Table 2:** Empathy scores between timepoints.

	T3 versus T0 JSE-S (*N* = 90)/ IRI (*N* = 88)			T2 versus T1 JSE-s (*N* = 88)/ IRI (*N* = 88)			T3 versus T2 JSE-s (*N* = 89)/ IRI (*N* = 88)		
	Mean diff. (CI)	t	p	Mean diff. (CI)	t	p	Mean diff. (CI)	t	p
**JSE-s Total**	7.23 (4.91 to 9.55)	6.19	< 0.001	2.28 (0.25 to 4.31)	2.23	0.028	5.05 (3.06 to 7.04)	5.05	< 0.001
**JSE-s PT**	1.84 (0.5 to 3.18)	2.73	0.008	0.39 (−0.78 to 1.58)	0,67	0.504	1.15 (−0.08 to 2.38)	1.86	0.067
**JSE-s CC**	3.75 (2.57 to 4.92)	6.34	< 0.001	1.29 (0.06 to 2.52)	2.08	0.041	2.75 (1.68 to 3.82)	5.11	< 0.001
**JSE-s STS**	1.64 (1.06 to 2.23)	5.54	< 0.001	0.59 (0.08 to 1.11)	2.31	0.023	1.15 (0.59 to 1.7)	4.08	< 0.001
**IRI Total**	7.08 (4.83 to 9.32)	6.27	< 0.001	−0.24 (−1.80 to 1.32)	−0.31	0.760	4.77 (2.96 to 6.58)	5.24	< 0.001
**IRI PT**	2.81 (1.84 to 3.78)	5.74	< 0.001	−0.22 (−0.98 to 0.55)	−0.56	0.575	1.66 (0.86 to 2.47)	4.10	< 0.001
**IRI FS**	2.05 (0.82 to 3.29)	3.32	0.001	0.24 (−0.44 to 0.93)	0.71	0.480	1.58 (0.58 to 2.59)	3.13	0.002
**IRI EC**	1.43 (0.66 to 2.19)	3.72	< 0.001	−0.28 (−0.88 to 0.32)	−0.93	0.357	1.15 (0.56 to 1.74)	3.88	< 0.001
**IRI PD**	0.68 (−0.25 to 1.61)	1.46	0.148	0.01 (−0.66 to 0.69)	0.04	0.969	0.38 (−0.37 to 1.12)	1.00	0.319

Analysis approach used t-test (t); Mean diff. = Mean difference; CI: confidence interval; significant p < 0.05.

T0: Baseline (beginning of first year); T1: Beginning of third year; T2: End of the third year (after the first clinical rotation); T3: End of final year of studies.

JSE-S: Jefferson Scale of Empathy-students; PT: Perspective Taking; CC: Compassionate Care; STS: Standing in the Patient’s Shoes.

IRI: Interpersonal Reactivity Index; PT: Perspective Taking; FS: Fantasy Scale; EC: Empathic Concern; PD: Personal Distress.

Across the timepoints, effect sizes were large for the increases in JSE-S Total scores and the IRI Total Scores; η²p = 0.21 and η²p = 0.20 respectively. For the subscales that showed an increase in scores, the effect sizes were moderate to large; JSE-S CC = 0.23, JSE-S STS = 0.15, IRI PT = 0.18, IRI FS = 0.09, and IRI EC = 0.09.

### Factors associated with students’ empathy (multivariate analyses)

The overall regression model for the JSE-S total between the time periods T3 and T0 (end of final year and beginning of first year) indicated that personality traits of neuroticism [mean difference (95% CI) = 0.51 (0.22 to 0.81), p = 0.001], extraversion [mean difference (95% CI) = 0.46 (0.15 to 0.76), p = 0.004], and agreeableness [mean difference (95% CI) = −0.5 (−0.99 to −0.01), p = 0.047] were associated with empathy scores. Similar results were found for the JSE-S CC subscale. Conscientiousness was also associated for the JSE-S STS subscale. All other factors were not associated (see S1 Table). In the IRI scale, conscientiousness was associated with the empathy scores in the IRI total [mean difference (95% CI) = −0.43 (−0.79 to −0.06), p = 0.021] and IRI FS subscale [mean difference (95% CI) = −0.23 (−0.43 to −0.03), p = 0.026]. Agreeableness was related to the IRI EC subscale [mean difference (95% CI) = −0.18 (−0.34 to −0.02)), p = 0.028]. Other variables were not associated with the IRI scores (see S2 Table).

Between T2 and T1 (end of the third year and the beginning of third year) – pre versus post the first clinical rotation – only “experience of a serious illness of self” [mean difference (95% CI) = −10.00 (−16.7 to −3.37), p = 0.004] was associated with the empathy score for JSE-S total and the JSE-S PT subscale (see S3 Table). “Openness” personality trait was associated with the empathy in the IRI total [mean difference (95% CI) = 0.34 (0.07 to 0.61), p = 0.014], and for the subscales IRI PT and IRI EC, while “Conscientiousness” was associated with empathy in the IRI PD subscale [mean difference (95% CI) = −0.13 (−0.25 to −0.02), p = 0.024]. Medical Specialty preferences was related to empathy in the IRI EC subscale [mean difference (95% CI) = −1.53 (−2.94 to −0.11), p = 0.035] (see S4 Table).

Between T2 and T3 (end of the third year and end of final year), the timepoints during which the “Medical Identity Journey” program was offered, only neuroticism was associated with changes in the JSE Total score (see S5 and S6 Tables). No associations were found with the IRI total and subscale scores.

### Baseline characteristics and scores

Except for the personality trait conscientiousness and gender, there were no statistically significant differences in demographic characteristics, JSE-S scores and IRI-scores at baseline between the group of participants who participated at all four timepoints versus those who did not (see S7 and S8 Tables). Indeed, students who provided JSE-S data at all four timepoints were more conscientiousness than those who did not (p = 0.010). Similar results were found for the students who provided IRI data at all four timepoints versus those who did not (p = 0.003). However, in our analyses, there was a minimal effect of this trait on student’s empathy when the timepoints T3 and T2 were compared (see S6 Table). There was a larger representation of females in the group that submitted IRI data at all four timepoints than those who did not (p = 0.044), but no significant differences were found related to gender when we analyzed the factors that could modify empathy between the timepoints (see S2, S4 and S6 Tables).

## Discussion

In this longitudinal, multi-year, prospective study of a cohort of undergraduate medical students at the University of Navarra (graduation class of 2022), participants were found to have higher empathy scores – as self-reported with the JSE-S and IRI instruments – at the time of graduation compared to the beginning of their studies six years previously. The curriculum includes an obligatory, multi-year intervention that includes workshops and written reflections during clinical rotations that are done from the fourth year of studies to the end of the final year (sixth year).

These results are encouraging as several studies have previously found no increases in empathy scores using instruments such as the JSE-S over the course of medical studies [28–30]. Others found an erosion of empathy [13–16,31–33]. A small number of studies showed maintenance or increase of empathy [11,12,17,34], but the interventions were generally brief. The study by Kötter et al., like ours, assessed empathy longitudinally across six years of medical training using the JSE-S and found a statistically significant increase in empathy over time [35].

In our study, empathy increased over the six years as assessed by the IRI instrument. We could not find other studies that used this instrument longitudinally for the purposes of comparison. Of interest in our study is that while the scores increased over the years for the IRI total score, there was no increase in the IRI PD subscale score. This is encouraging as it suggests an increase in empathy without increasing personal distress. Decety and Meyer [36] and Airagnes and colleagues [37] have highlighted the importance of self-regulation and self-awareness while nurturing empathy so as not to cause, among others, burnout. It is possible that our “Medical Identity Itinerary” curriculum, given an emphasis on self-reflection as part of the process, may have a protective impact [38].

We found significant increases in empathy as measured by the JSE-S instrument (total and two subscales) following the first clinical rotation (T2-T1). However, no impact was noted as measured by the IRI instrument. This may be explained by a key difference between the two instruments; the JSE-S´ items are stated in the third person and it assesses mainly the cognitive component of empathy, while the IRI´s statements are in the first person and could therefore be perceived to be more personal. There may be a social desirability bias at play in which students prefer not to describe themselves negatively. It is also possible that the only one short clinical rotation (1 month) during that time period is not sufficient to impact empathy.

The increase in some of the empathy scores in the time intervals preceding the start of the Medical Identity Journey (fourth year) merits some discussion. Increases were noted in the IRI total and three of its four subscales between timeperiods T0 and T1 (start of the first year and beginning of the third year). From the first to the third year, students take subjects such as Introduction to Clinical Practice, where they learn general principles of professionalism in the clinical interview and participate in workshops with standardized patients, as well as Anthropology, Psychology, Keys to Contemporary Culture, and Ethics. These could have contributed to these increases. For the JSE-S, increases were noted in the JSE-S total and subscale scores between timeperiods T1 and T2 (beginning of the third year and end of the third year). During the third year, students start their first clinical rotation. This could explain, at least partially, the increase in score during this timeline.

The literature highlights significant associations between personality traits and empathy, and that personality traits are associated with outcomes in medical education and medical practice [39–46]. Costa et al, for example, found that the traits of agreeableness and openness were associated with more empathy [41]. Guilera et al. confirmed a relationship between empathy and personality using three different measures of empathy [44]. Betkowska-Karpala and colleagues found three different clusters or profiles of medical students’ empathy and there were important differences between these profiles in various indicators of empathy and personality [47]. They propose that awareness of this gives an opportunity to better adjust and profile training techniques, as well as inform choices regarding future medical specialization. Moreover, because each empathy profile is manifested in relations with patients in specific ways, medical education in empathy holds great potential to reduce anxiety, stress, and burnout associated with the medical profession; students may be taught how their empathy and personality characteristics may influence their everyday medical practice [47].

In our study, the personality traits neuroticism, extraversion and agreeableness were associated with empathy changes between the end of the final year and beginning of the first year in the JSE-S total score, and conscientiousness in the case of the IRI total score. However, these associations, except for neuroticism, disappear when we explored associations between the end of the final year and the end of the third year. This may, as others have also found, highlight the importance of the first years of medicine training in terms of nurturing empathy. Students with higher levels of neuroticism tended to exhibit an increase in empathy over the course of the degree, as reflected in the total JSE-S score. Neuroticism is characterized by a tendency to experience negative emotions and heightened self‐consciousness, which may foster the development of greater empathy towards others.

Personality is a complex construct in terms of its relationship to medical education. Although personality traits are generally considered relatively stable over the course of medical training, some individuals do experience changes in some personality traits [48]. In our study we measured students’ personality only at the beginning of first year and are therefore unable to study the influence of personality stability or change over the course of training.

Similarly to other researchers, our study found that specialty preferences, gender, volunteer experience, and the number of rotations were not associated with increased empathy as assessed by the JSE-S [32,49,50]. However, Lwow and colleagues, also using the JSE-S, did find greater empathy scores amongst women than men [51]. We did identify higher values of baseline empathy amongst women in the IRI scale (mainly in the affective component: IRI EC and IRI PD). This is aligned with the findings of other studies [25,44,52]. Encouragingly, in our study, empathy increased across genders.

We have intentionally incorporated a strategy to enhance medical identity in our curriculum through the program described herein. The results of this study, and our previous study, provide us some assurance that we are on the right track. Intentional strategies are needed to nurture the growth of empathy and compassion across medical training and to mitigate factors that undermine them [53]. Eikeland and colleagues, for example, found that empathy and compassion can be eroded by medical students’ priority to master biomedical knowledge over what is perceived to be “soft skills”, a perceived need to retain emotional control and to ensure emotional distancing from patients (especially in the case of challenging situations such as suffering, death or medical error), and cynicism as a coping strategy to deal with perceived time pressures and heavy workloads [54]. Hidden curricula, including assessment methods, that prioritize knowledge and interventional skills over patient-centeredness may accentuate these perceptions and undermine empathy and compassion [55].

A recent review by Zhou and colleagues describes the various learning approaches used to nurture empathy [11]. Our program “Medical Identity Journey” uses several of these, including workshops that incorporate the humanities and case-based learning, and personal written reflections based on experiences during the clinical rotations. In addition to this program, two other courses whose goals include nurturing empathy, compassion and person-centred care, are included in the medical curriculum; an obligatory palliative care course in the sixth year that includes simulated patients and an optional week-long palliative care course [56].

While our study was not designed to study the individual impact of each of the curriculum components (clinical rotations, workshops, reflective portfolios) on empathy scores, the changes in the two instruments and the timepoints when those improvements occurred suggest that together, as a collective, the components have an effect. In a previous cross-sectional study comparing two cohorts of final year students, we found higher levels of empathy as assessed by standardized patients amongst student that received the curriculum versus a control group [19]. The impact of clinical rotations on empathy and compassion is still unclear [32,57–59]. Role modeling by clinical supervisors during the rotations may play a role [60–62]. Some clinical rotation settings may nurture empathy more than others [59]. The impact of empathy-related self-reflection portfolios by medical students during clerkship is also not clear [63]. These three questions related to nurturing empathy in medical education – the specific role of clinical rotations and if so which ones, and the role of role-modeling – present opportunities for future research in this area,

A strength of our study was the use of two instruments simultaneously to assess empathy, and these were administered longitudinally at different time points. The two instruments are complementary in that the IRI focuses on measures of both cognitive and affective empathy while the JSE-S highlights the cognitive domain, and Zhou et al. have proposed the use of more than one instrument when exploring empathy [11]. In addition to exploring statistical differences, we also calculated effect sizes to determine how large and meaningful differences were; these showed moderate to large effect sizes, which reinforces the robustness of our conclusions.

Several limitations are recognized. Attrition of participants occurred across the four timepoints. By analyzing only those who completed data at all four timepoints, an attrition bias may have occurred, and our approach runs the risk, among others, of students with low empathy possibly being less likely to complete the forms over time. Linear mixed models (LMM), as used by Carrard et al. for example, may better address missing data in these longitudinal studies [34]. However, our finding that the baseline scores between the students who participated at all four timepoints and those who did not were statistically similar (except for the conscientiousness trait) provides some support to our assumption that the data are missing completely at random (MCAR) and therefore analyzing only those that submitted data at all four timepoints is a reasonable approach. The study did not include a comparison group which could have helped explore the contribution of the Medical Identity Journey program relative to the whole curriculum and the impact of the curriculum in the first to third years of study. Lastly, the study focused on aspects of empathy, and we did not explore the other aspects of medical identity such as professionalism, ethical decision-making and teamwork.

## Conclusion

In this longitudinal, multi-year study of a class of medical students who graduated in 2022, we found significant increases in empathy, as measured by the JSE-S and IRI instruments, from the beginning of their studies to the end of the final year six years later. From a patient-centered care approach, this is encouraging. The six-year medical school curriculum includes a mandatory program from the fourth year of training to the final year of training that aims to nurture empathy. This multi-strategy program includes workshops on themes such as empathy and compassion prior to each clinical rotations from year’s fourth to six of studies, and written reflections on these themes following each of the clinical rotations using a portfolio approach.

Other aspects of medical identity are incorporated in the program, including professionalism, ethical decision-making and teamwork. The impact of the program on these domains merits future research, as does the relative contributions of each of the program’s components.

## Supporting information

S1 Table Change in Jefferson Scale of Empathy for Medical Students (JSE-S) and its subscale scores between T3 (end of year 6) and T0 (baseline) by different factors related to empathy (*N* = 88). (DOCX)

S2 Table Change in Interpersonal Reactivity Index (IRI) and its subscale scores between T3 (end of year 6) and T0 (baseline) by different factors related to empathy (*N* = 90). (DOCX)

S3 Table Change in Jefferson Scale of Empathy for Medical Students (JSE-S) and its subscale scores between T2 and T1 (pre versus post the first clinical rotation, year 3) by different factors related to empathy (N = 88). (DOCX)

S4 Table Change in Interpersonal Reactivity Index (IRI) and its subscale scores between T2 and T1 (pre versus post the first clinical rotation, year 3) by different factors related to empathy (N = 88). (DOCX)

S5 Table Change in Jefferson Scale of Empathy for Medical Students (JSE-S) and its subscale scores between T3 (end of final year of studies) and T2 (end of the third year) by different factors related to empathy (N = 88). (DOCX)

S6 Table Change in Interpersonal Reactivity Index (IRI) and its subscale scores between T3 (end of final year of studies and T2 (end of the third year) by different factors related to empathy (N = 89). (DOCX)

S1 Data base aug 2025. (XLSX)
