# Supplementary material for: Increase in self-reported empathy during medical school training: A longitudinal study
Source: PLoS One. 2025 Sep 15;20(9):e0332343. doi: 10.1371/journal.pone.0332343 (PMC12435721; doi:10.1371/journal.pone.0332343)
Supplement: S1 Table — (DOCX) [file pone.0332343.s001.docx]

S1 Table. Change in Jefferson Scale of Empathy for Medical Students (JSE-S) and its subscale scores between T3 (end of year 6) and T0 (baseline) by different factors related to empathy (*N* = 90).

|  | **JSE-S Total** | | **JSE-S PT** | | **JSE-S CC** | | | | **JSE-S STS** | |
| --- | --- | --- | --- | --- | --- | --- | --- | --- | --- | --- |
|  | **Mean diff.**  **(95% CI)** | **p value** | **Mean diff.**  **(95% CI)** | **P value** | **Mean diff.**  **(95% CI)** | **p value** | | | **Mean diff.**  **(95% CI)** | **p value** |
| **Gender**: Male vs female | -0.32 (-5.52 to 4.89) | 0.904 | 0.51 (-2.75 to 2.85) | 0.971 | -0.14 (-3.13 to 3.1) | | 0.990 | | -0.35 (-1.7 to 0.99) | 0.605 |
| **Own serious illness**: yes vs no | 2.28 (-7.13 to 11.69) | 0.630 | -0.23 (-5.28 to 4.83) | 0.929 | 3.47 (-2.16 to 9.1) | | 0.223 | | -0.96 (-3.41 to 1.48) | 0.434 |
| **Serious illness of someone close**: yes vs no | 4.73 (-2.19 to 11.65) | 0.177 | 1.16 (-2.55 to 4.87) | 0.535 | 1.88 (-2.26 to 6.02) | | 0.368 | | 1.69 (-0.1 to 3.49) | 0.065 |
| **Volunteerism**: yes vs no | 0.78 (-6.39 to 7.95) | 0.829 | -1.76 (-5.61 to 2.09) | 0.365 | 2.55 (-1.74 to 6.84) | | 0.240 | | -0.01 (-1.87 to 1.85) | 0.992 |
| **Personality:** |  | |  |  |  | | |  | | |
| Neuroticism | 0.51 (0.22 to 0.81) | 0.001 | 0.22 (0.06 to 0.38) | 0.006 | 0.22 (0.46 to 0.39) | | 0.014 | | 0.07 (-0.01 to 0.15) | 0.065 |
| Extraversion | 0.46 (0.15 to 0.76) | 0.004 | 0.16 (-0.01 to 0.32) | 0.064 | 0.24 (0.05 to 0.42) | | 0.013 | | 0.06 (-0.02 to 0.14) | 0.118 |
| Openness | -0.05 (-0.38 to 0.29) | 0.779 | -0.01 (-0.19 to 0.17) | 0.931 | -0.07 (-0.27 to 0.13) | | 0.476 | | 0.03 (-0.05 to 0.12) | 0.459 |
| Agreeableness | -0.5 (-0.99 to -0.01) | 0.047 | -0.1 (-0.37 to 0.16) | 0.445 | -0.31 (-0.6 to -0.02) | | 0.040 | | -0.09 (-0.22 to 0.04) | 0.172 |
| Conscientiousness | 0.36 (-0.01 to 0.73) | 0.058 | 0.05 (-0.15 to 0.25) | 0.619 | 0.17 (-0.05 to 0.39) | | 0.138 | | 0.14 (0.045 to 0.24) | 0.005 |
| **Specialty preference**  Non-medical vs medical | -1.53 (-8.32 to 5.25) | 0.654 | -0.01 (-3.65 to 3.64) | 0.997 | -0.73 (-4.79 to 3.33) | | 0.721 | | -0.79 (-2.56 to 0.97) | 0.371 |
| **Medical internships**  < 5 medical specialty vs ≥ 5 | -2.82 (-8.19 to 2.54) | 0.298 | -0.72 (-3.61 to 2.16) | 0.618 | -1.49 (-4.71 to 1.71) | | 0.356 | | -0.6 (-1.99 to 0.79) | 0.393 |

Mean diff.: Mean difference; CI: confidence interval; significant p < 0.05.

JSE-S: Jefferson Scale of Empathy-students; PT: Perspective Taking; CC: Compassionate Care; STS: Standing in the Patient’s Shoes.
