# Supplementary material for: Increase in self-reported empathy during medical school training: A longitudinal study
Source: PLoS One. 2025 Sep 15;20(9):e0332343. doi: 10.1371/journal.pone.0332343 (PMC12435721; doi:10.1371/journal.pone.0332343)
Supplement: S2 Table — (DOCX) [file pone.0332343.s002.docx]

S2 Table. Change in Interpersonal Reactivity Index (IRI) and its subscale scores between T3 (end of year 6) and T0 (baseline) by different factors related to empathy (*N* = 88).

|  | **IRI Total** | | **IRI PT** | | **IRI FS** | | **IRI EC** | | **IRI PD** | |
| --- | --- | --- | --- | --- | --- | --- | --- | --- | --- | --- |
|  | **Mean diff.**  **(95% CI)** | **p**  **value** | **Mean diff.**  **(95% CI)** | **p**  **value** | **Mean diff.**  **(95% CI)** | **p value** | **Mean diff.**  **(95% CI)** | **p value** | **Mean diff.**  **(95% CI)** | **p**  **value** |
| **Gender**: Male vs female | -3.38 (-8.47 to 1.72) | 0.191 | 0.73 (-1.53 to 2.99) | 0.522 | -0,20 (-3.01 to 2.62) | 0.889 | -1.80 (-3.49 to -0.11) | 0.038 | -5.06 (-4.24 to 0.13) | 0.064 |
| **Own serious illness**: yes vs no | 0.43 (-8.79 to 9.64) | 0.927 | -1.77 (-5.86 to 2.32) | 0.392 | 3.79 (-1.31 to 8.88) | 0.134 | -0.70 (-3.76 to 2.36) | 0.651 | -0.71 (-4.66 to 3.24) | 0.720 |
| **Serious illness of someone close**: yes vs no | -3.96 (-10.73 to 2.81) | 0.247 | -1.87 (-4.88 to 1.13) | 0.219 | -1.37 (-5.11 to 2.37) | 0.469 | 0.89 (-1.38 to 3.14) | 0.434 | -1.54 (-4.47 to 1.36) | 0.292 |
| **Volunteerism**: yes vs no | 5.19 (-1.83 to 12.21) | 0.145 | -1.06 (-4.17 to 2.06) | 0.501 | 3.48 (-0.40 to -7.36) | 0.078 | 2.25 (-0.08 to 4.58) | 0.058 | 0.44 (-2.56 to 3.46) | 0.767 |
| **Personality** |  |  |  |  |  |  |  |  |  |  |
| Neuroticism | -0.03 (-0.32 to 0.26) | 0.836 | 0.02 (-0.11 to 0.14) | 0.799 | 0.03 (-0.13 to 0.19) | 0.719 | -0.02 (-0.12 to 0.07) | 0.595 | -0.07 (-0.19 to 0.06) | 0.282 |
| Extraversion | -0.03 (-0.33 to 0.27) | 0.861 | 0.07 (-0.06 to 0.21) | 0.283 | 0.04 (-0.13 to 0.20) | 0.671 | -0.06 (-0.16 to 0.04) | 0.222 | -0.07 (-0.20 to 0.06) | 0.288 |
| Openness | -0.15 (-0.48 to 0.18) | 0.373 | -0.04 (-0.19 to 0.10) | 0.573 | -0.04 (-0.22 to 0.14) | 0.656 | -0.01 (-0.12 to 0.09) | 0.789 | -0.07 (-0.21 to 0.07) | 0.326 |
| Agreeableness | -0.44 (-0.92 to -0.04) | 0.074 | -0.13 (-0.34 to 0.08) | 0.247 | -0.19 (-0.46 to 0.08) | 0.160 | -0.18 (-0.34 to -0.02) | 0.028 | 0.08 (-0.12 to 0.29) | 0.434 |
| Conscientiousness | -0.43 (-0.79 to -0.06) | 0.021 | -0.07 (-0.23 to 0.09) | 0.370 | -0.23 (-0.43 to -0.03) | 0.026 | -0.10 (-0.22 to -0.02) | 0.096 | -0.04 (-0.20 to 0.11) | 0.562 |
| **Specialty preferences**  Non-medical vs medical | 1.49 (-5.16 to 8.13) | 0.657 | 1.64 (-1.31 to 4.59) | 0.270 | 1.75 (-1.92 to 5.43) | 0.344 | -0.65 (-2.85 to 1.56) | 0.560 | -1.68 (-4.52 to 1.17) | 0.244 |
| **Medical internships**  < 5 medical specialty vs ≥ 5 | -2.17 (-7.42 to 3.08) | 0.413 | -0.17 (-2.51 to 2.16) | 0.844 | -1.08 (-3.98 to 1.83) | 0.462 | -1.14 (-2.88 to 0.61) | 0.198 | 0.17 (-2.08 to 2.43) | 0.877 |

Mean diff.: Mean difference; CI: confidence interval; significant p < 0.05.

IRI: Interpersonal Reactivity Index; PT: Perspective Taking; FS: Fantasy Scale; EC: Empathic Concern; PD: Personal Distress.
