# Supplementary material for: Increase in self-reported empathy during medical school training: A longitudinal study
Source: PLoS One. 2025 Sep 15;20(9):e0332343. doi: 10.1371/journal.pone.0332343 (PMC12435721; doi:10.1371/journal.pone.0332343)
Supplement: S3 Table — (DOCX) [file pone.0332343.s003.docx]

S3 Table. Change in Jefferson Scale of Empathy for Medical Students (JSE-S) and its subscale scores between T2 and T1 (pre versus post the first clinical rotation, year 3) by different factors related to empathy (*N* = 88).

|  | **JSE-S Total** | | **JSE-S PT** | | | **JSE-S CC** | | **JSE-S STS** | |
| --- | --- | --- | --- | --- | --- | --- | --- | --- | --- |
|  | **Mean diff.**  **(95% CI)** | **p value** | **Mean diff.**  **(95% CI)** | **p value** | | **Mean diff.**  **(95% CI)** | **p value** | **Mean diff.**  **(95% CI)** | **p value** |
| **Gender**: Male vs female | 1.76 (-2.85 to 6.36) | 0.449 | -0.05 (-2.94 to 2.85) | | 0.974 | 1.98 (-0.89 to 4.85) | 0.172 | -0.18 (-1.39 to 1.03) | 0.768 |
| **Own serious illness**: yes vs no | -10.0 (-16.7 to -3.37) | 0.004 | -5.5 (-9.7 to -1.32) | | 0.011 | -3.09 (-7.25 to 1.06) | 0.141 | -1.43 (-3.17 to 0.32) | 0.108 |
| **Serious illness of someone close**: yes vs no | 2.02 (-3.41 to 7.46) | 0.460 | 0.31 (-3.11 to 3.73) | | 0.857 | 2.32 (-1.07 to 5.7) | 0.177 | -0.6 (-2.03 to 0.82) | 0.402 |
| **Volunteerism**: yes vs no | 0.64 (-5.89 to 7.16) | 0.847 | 1.07 (-3.03 to 5.18) | | 0.603 | -0.16 (-4.22 to 3.9) | 0.938 | -0.28 (-1.99 to 1.43) | 0.747 |
| **Personality** |  | |  | | |  | |  | |
| Neuroticism | 0.23 (-0.06 to 0.53) | 0.118 | 0.09 (-0.086 to 0.28) | | 0.290 | 0.15 (-0.04 to 0.33) | 0.116 | -0.01 (-0.09 to 0.07) | 0.764 |
| Extraversion | 0.17 (-0.12 to 0.46) | 0.241 | 0.03 (-0.15 to 0.21) | | 0.739 | 0.15 (-0.03 to 0.33) | 0.102 | -0.01 (-0.08 to 0.07) | 0.820 |
| Openness | -0.11 (-0.45 to 0.24) | 0.543 | 0.03 (-0.19 to 0.24) | | 0.814 | -0.08 (-0.29 to 0.14) | 0.470 | -0.53 (-0.14 to 0.04) | 0.246 |
| Agreeableness | -0.25 (-0.69 to 0.2) | 0.273 | 0.09 (-0.19 to 0.37) | | 0.538 | -0.23 (-0.51 to 0.05) | 0.106 | -0.11 (-0.23 to 0.01) | 0.074 |
| Conscientiousness | -0.08 (-0.43 to 0.28) | 0.661 | -0.13 (-0.35 to 0.09) | | 0.263 | 0.02 (-0.19 to 0.25) | 0.824 | 0.02 (-0.07 to 0.12) | 0.621 |
| **Specialty preference**  Non-medical vs medical | -0.82 (-5.89 to 4.25) | 0.749 | 1.09 (-2.09 to 4.28) | | 0.498 | -0.62 (-3.78 to 2.54) | 0.697 | -1.29 (-2.62 to 0.04) | 0.057 |
| **Medical internship:**  Non-Medical vs medical | -0.96 (-5.32 to 3.39) | 0.660 | -0.29 (-3.03 to 2.45) | | 0.832 | -0.75 (-3.46 to 1.97) | 0.585 | 0.08 (-1.07 to 1.22) | 0.896 |

Mean diff.: Mean difference; CI: confidence interval; significant p < 0.05.

JSE-S: Jefferson Scale of Empathy-students; PT: Perspective Taking; CC: Compassionate Care; STS: Standing in the Patient’s Shoes.
