# Supplementary material for: Increase in self-reported empathy during medical school training: A longitudinal study
Source: PLoS One. 2025 Sep 15;20(9):e0332343. doi: 10.1371/journal.pone.0332343 (PMC12435721; doi:10.1371/journal.pone.0332343)
Supplement: S4 Table — (DOCX) [file pone.0332343.s004.docx]

S4 Table. Change in Interpersonal Reactivity Index (IRI) and its subscale scores between T2 and T1 (pre versus post the first clinical rotation, year 3) by different factors related to empathy (*N* = 88).

|  | **IRI Total** | | **IRI PT** | | **IRI FS** | | **IRI EC** | | **IRI PD** | |
| --- | --- | --- | --- | --- | --- | --- | --- | --- | --- | --- |
|  | **Mean diff.**  **(95% CI)** | **p**  **value** | **Mean diff.**  **(95% CI)** | **p**  **value** | **Mean diff.**  **(95% CI)** | **p value** | **Mean diff.**  **(95% CI)** | **p value** | **Mean diff.**  **(95% CI)** | **p**  **value** |
| **Gender**: Male vs female | -1.33 (-4.96 to 2.29) | 0.464 | 1.32 (-0.49 to 3.11) | 0.149 | -0.87 (-2.59 to 0.85) | 0.317 | -0.93 (-2.27 to 0.41) | 0.171 | -0.85 (-2.40 to 0.70) | 0.277 |
| **Own serious illness**: yes vs no | 1.48 (-3.51 to 6.46) | 0.556 | 0.92 (-1.55 to 3.40) | 0.459 | 0.85 (-1.52 to 3.22) | 0.478 | 1.24 (-0.61 to 3.09) | 0.185 | -1.53 (-3.67 to 0.60) | 0.156 |
| **Serious illness of someone close**: yes vs no | -1.14 (-5.19 to 2.91) | 0.576 | -1.46 (-3.47 to 0.56) | 0.153 | 0.20 (-1.73 to 2.12) | 0.840 | 0.61 (-0.89 to 2.12) | 0.419 | -0.49 (-2.23 to 1.24) | 0.574 |
| **Volunteerism**: yes vs no | -2.00 (-6.91 to 2.91) | 0.419 | -1.12 (-3.56 to 1.32) | 0.363 | -0.94 (-3.28 to 1.39) | 0.423 | 0.06 (-1.76 to 1.88) | 0.950 | 0.01 (-2.10 to 2.11) | 0.995 |
| **Personality** |  |  |  |  |  |  |  |  |  |  |
| Neuroticism | -0.21 (-0.42 to 0.01) | 0.057 | -0.08 (-0.18 to 0.03) | 0.145 | 0.00 (-0.10 to 0.10) | 0.938 | -0.05 (-0.13 to 0.03) | 0.194 | -0.08 (-0.17 to 0.01) | 0.083 |
| Extraversion | 0.19 (-0.03 to 0.42) | 0.095 | -0.00 (-0.12 to 0.11) | 0.943 | 0.10 (-0.00 to 0.21) | 0.054 | 0.04 (-0.04 to 0.12) | 0.340 | 0.05 (-0.05 to 0.16) | 0.308 |
| Openness | 0.34 (0.07 to 0.61) | 0.014 | 0.16 (0.03 to 0.30) | 0.016 | 0.06 (-0.06 to 0.19) | 0.323 | 0.12 (0.02 to 0.22) | 0.020 | -0.01 (-0.12 to 0.11) | 0.880 |
| Agreeableness | -0.02 (-0.36 to 0.32) | 0.914 | 0.03 (-0.13 to 0.20) | 0.694 | -0.07 (-0.23 to 0.09) | 0.395 | 0.05 (-0.08 to 0.17) | 0.459 | -0.03 (-0.17 to 0.11) | 0.685 |
| Conscientiousness | -0.16 (-0.42 to 0.11) | 0.254 | -0.04 (-0.18 to 0.09) | 0.508 | 0.12 (-0.01 to 0.25) | 0.062 | -0.10 (-0.20 to 0.00) | 0.052 | -0.13 (-0.25 to -0.02) | 0.024 |
| **Specialty preferences**  Non-medical vs medical | -3.34 (-7.16 to 0.47) | 0.085 | -0.35 (-2.24 to 1.55) | 0.715 | -1.10 (-2.91 to 0.72) | 0.232 | -1.53 (-2.94 to -0.11) | 0.035 | -0.37 (-2.00 to 1.27) | 0.654 |
| **Medical internships:**  No-Medical vs medical | 2.77 (-0.61 to 6.15) | 0.106 | 1.51 (-0.17 to 3.20) | 0.077 | 0.11 (-1.50 to 1.71) | 0.896 | 0.61 (-0.65 to 1.86) | 0.336 | 0.54 (-0.91 to 1.99) | 0.458 |

Mean diff.: Mean difference; CI: confidence interval; significant p < 0.05.

IRI: Interpersonal Reactivity Index; PT: Perspective Taking; FS: Fantasy Scale; EC: Empathic Concern; PD: Personal Distress.
