# Supplementary material for: Increase in self-reported empathy during medical school training: A longitudinal study
Source: PLoS One. 2025 Sep 15;20(9):e0332343. doi: 10.1371/journal.pone.0332343 (PMC12435721; doi:10.1371/journal.pone.0332343)
Supplement: S5 Table — (DOCX) [file pone.0332343.s005.docx]

S5 Table. Change in Jefferson Scale of Empathy for Medical Students (JSE-S) and its subscale scores between T3 (end of final year of studies) and T2 (end of the third year) by different factors related to empathy (*N* = 89).

|  | **JSE-S Total** | | **JSE-S PT** | | **JSE-S CC** | | **JSE-S STS** | |
| --- | --- | --- | --- | --- | --- | --- | --- | --- |
|  | **Mean diff.**  **(95% CI)** | **p value** | **Mean diff.**  **(95% CI)** | **p value** | **Mean diff.**  **(95% CI)** | **p value** | **Mean diff.**  **(95% CI)** | **p value** |
| **Gender**: Male vs female | 3.24 (-1.85 to 8.32) | 0.208 | 2.29 (-0.33 to 4.93) | 0.086 | 1.29 (-1.90 to 4.49) | 0.423 | -0.35 (-1.94 to 1.24) | 0.661 |
| **Own serious illness**: yes vs no | 3.58 (-6.93 to 14.08) | 0.499 | -1.78 (-7.22 to 3.66) | 0.516 | 3.71 (-2.90 to 10.32) | 0.266 | 1.65 (-1.64 to 4.93) | 0.321 |
| **Serious illness of someone close**: yes vs no | 0.69 (-4.32 to 5.71) | 0.781 | 1.24 (-1.35 to 3.84) | 0.342 | -1.29 (-4.44 to 1.87) | 0.418 | 0.74 (-0.83 to 2.30) | 0.349 |
| **Volunteerism**: yes vs no | -0.34 (-6.04 to 5.36) | 0.905 | -0.77 (-1.72 to 2.18) | 0.604 | 0.5 (-3.08 to 4.09) | 0.779 | -0.07 (-1.86 to 1.70) | 0.933 |
| **Personality** |  | |  | |  | |  | |
| Neuroticism | 0.30 (0.02 to 0.59) | 0.034 | 0.21 (0.07 to 0.36) | 0.005 | 0.08 (-0.10 to 0.26) | 0.379 | 0.01 (-0.07 to 0.09) | 0.790 |
| Extraversion | 0.15 (-0.13 to 0.44) | 0.291 | 0.07 (-0.08 to 0.22) | 0.339 | 0.11 (-0.71 to 0.29) | 0.234 | -0.03 (-0.12 to 0.61) | 0.547 |
| Openness | 0.09 (-0.24 to 0.43) | 0.580 | 0.05 (-0.12 to 0.22) | 0.574 | 0.06 (-0.15 to 0.27) | 0.577 | -0.02 (-0.12 to 0.89) | 0.777 |
| Agreeableness | -0.26 (-0.75 to 0.23) | 0.288 | -0.01 (-0.26 to 0.25) | 0.974 | -0.19 (-0.49 to 0.11) | 0.216 | -0.07 (-0.22 to 0.87) | 0.394 |
| Conscientiousness | 0.38 (-0.00 to 0.77) | 0.051 | 0.10 (-0.10 to 0.30) | 0.302 | 0.23 (-0.01 to 0.48) | 0.058 | 0.05 (-0.07 to 0.17) | 0.448 |
| **Specialty preference**  Non-medical vs medical | -3.36 (-9.89 to 2.77) | 0.278 | 0.74 (-2.44 to 3.91) | 0.645 | -3.03 (-6.89 to 0.83) | 0.121 | -1.06 (-2.98 to 0.85) | 0.271 |
| **Medical internships:**  < 5 medical specialty vs ≥ 5 | -0.52 (-5.59 to 4.55) | 0.838 | -0.29 (-2.92 to 2.34) | 0.826 | 0.46 (-2.73 to 3.66) | 0.772 | -0.70 (-2.28 to 0.89) | 0.384 |

Mean diff.: Mean difference; CI: confidence interval; significant p < 0.05.

JSE-S: Jefferson Scale of Empathy-students; PT: Perspective Taking; CC: Compassionate Care; STS: Standing in the Patient’s Shoes.
