# Supplementary material for: Increase in self-reported empathy during medical school training: A longitudinal study
Source: PLoS One. 2025 Sep 15;20(9):e0332343. doi: 10.1371/journal.pone.0332343 (PMC12435721; doi:10.1371/journal.pone.0332343)
Supplement: S6 Table — (DOCX) [file pone.0332343.s006.docx]

S6 Table. Change in Interpersonal Reactivity Index (IRI) and its subscale scores between T3 (end of final year of studies) and T2 (end of the third year) by different factors related to empathy (*N* = 88).

|  | **IRI Total** | | **IRI PT** | | **IRI FS** | | **IRI EC** | | **IRI PD** | |
| --- | --- | --- | --- | --- | --- | --- | --- | --- | --- | --- |
|  | **Mean diff.**  **(95% CI)** | **p**  **value** | **Mean diff.**  **(95% CI)** | **p**  **value** | **Mean diff.**  **(95% CI)** | **p value** | **Mean diff.**  **(95% CI)** | **p value** | **Mean diff.**  **(95% CI)** | **p**  **value** |
| **Gender**: Male vs female | 1.75 (-3.05 to 6.55) | 0.468 | 0.35 (-1.63 to 2.33) | 0.726 | 1.36 (-1.42 to 4.14) | 0.333 | 0.31 (-1.28 to 1.89) | 0.702 | -0.26 (-2.30 to 1.77) | 0.797 |
| **Own serious illness**: yes vs no | 2.91 (-6.88 to 12.71) | 0.554 | 1.63 (-2.42 to 5.68) | 0.423 | -1.25 (-6.93 to 4.44) | 0.662 | 0.83 (-2.41 to 4.07) | 0.609 | 1.70 (-2.46 to 5.85) | 0.417 |
| **Serious illness of someone close**: yes vs no | 0.46 (-4.29 to 5.22) | 0.847 | 0.56 (-1.41 to 2.52) | 0.572 | -0.18 (-2.94 to 2.58) | 0.896 | -0.89 (-2.46 to 0.69) | 0.264 | 0.97 (-1.04 to 2.99) | 0.338 |
| **Volunteerism**: yes vs no | 1.03 (-4.29 to 6.35) | 0.700 | -2.02 (-4.22 to 0.18) | 0.206 | 2.81 (-0.28 to 5.90) | 0.073 | 0.25 (-1.51 to 2.01) | 0.780 | -0.01 (-2.26 to 2.25) | 0.996 |
| **Personality** |  |  |  |  |  |  |  |  |  |  |
| Neuroticism | 0.11 (-0.15 to 0.37) | 0.417 | 0.05 (-0.06 to 0.15) | 0.412 | -0.04 (-0.19 to 0.11) | 0.627 | 0.01 (-0.07 to 0.10) | 0.786 | 0.09 (-0.02 to 0.20) | 0.120 |
| Extraversion | 0.05 (-0.22 to 0.31) | 0.721 | 0.07 (-0.04 to 0.18) | 0.217 | -0.02 (-0.17 to 0.13) | 0.778 | -0.04 (-0.12 to 0.05) | 0.407 | 0.04 (-0.07 to 0.15) | 0.505 |
| Openness | -0.29 (-0.61 to 0.02) | 0.067 | -0.10 (-0.23 to 0.03) | 0.115 | -0.05 (-0.23 to 0.13) | 0.585 | -0.08 (-0.18 to 0.02) | 0.135 | -0.06 (-0.19 to 0.07) | 0.367 |
| Agreeableness | -0.19 (-0.64 to 0.27) | 0.417 | -0.10 (-0.28 to 0.09) | 0.310 | -0.12 (-0.39 to 0.14) | 0.349 | -0.06 (-0.21 to 0.09) | 0.464 | 0.09 (-0.10 to 0.28) | 0.353 |
| Conscientiousness | -0.03 (-0.39 to 0.33) | 0.859 | -0.01 (-0.15 to 0.14) | 0.934 | -0.12 (-0.33 to 0.08) | 0.243 | 0.05 (-0.07 to 0.16) | 0.436 | 0.05 (-0.10 to 0.20) | 0.509 |
| **Specialty preferences**  Non-medical vs medical | -0.09 (-5.79 to 5.62) | 0.976 | -1.51 (-3.87 to 0.85) | 0.206 | 1.77 (-1.54 to 5.08) | 0.290 | -0.27 (-2.16 to 1.61) | 0.772 | -0.07 (-2.49 to 2.35) | 0.955 |
| **Medical internships**  < 5 medical specialty vs ≥ 5 | -1.99 (-6.72 to 2.73) | 0.402 | 0.43 (-1.52 to 2.39) | 0.660 | -0.73 (-3.47 to 2.01) | 0.597 | -0.42 (-1.98 to 1.15) | 0.596 | -1.28 (-3.28 to 0.72) | 0.206 |

Mean diff.: Mean difference; CI: confidence interval; significant p < 0.05.

IRI: Interpersonal Reactivity Index; PT: Perspective Taking; FS: Fantasy Scale; EC: Empathic Concern; PD: Personal Distress.
